# Resistance of External Thermal Insulation Composite Systems with Rendering (ETICS) to Hail

**DOI:** 10.3390/ma13112452

**Published:** 2020-05-28

**Authors:** Barbara Francke, Renata Zamorowska

**Affiliations:** Instytut Techniki Budowlanej, Filtrowa 1, 00-611 Warsaw, Poland; r.zamorowska@itb.pl

**Keywords:** thermal insulation systems, reinforced layer, hail resistance, fracture toughness

## Abstract

This paper analyzes the resistance to hail of external thermal insulation composite systems (ETICS), i.e., external thermal insulation of foamed polystyrene with the same finishing coat and various reinforcing mesh and base coats used to make the reinforced layer. The manuscript presents our own new method for assessing ETICS resistance to hail and test results obtained according to this method. The basic premise of the presented new research methodology is evaluation of the thermal insulation system surface damage and fracture toughness, in the function of hit velocity with a polyamide ball with a standardized diameter and weight. The results of hail resistance tests were compared with the values of hard body impact resistance obtained in the tests done according to ETAG 004. Results obtained by the new method help to evaluate precisely the resistance of thermal insulation sets to damage as a result of impact of heavy objects of permanent shape, with greater accuracy than the hard body impact test. They also confirmed that thermal insulation sets with dispersion adhesive in the reinforcement demonstrate greater resistance to damage as a result of hail impact than the sets with cement-based adhesives and that weight of the reinforcing mesh used in the system is not significant to affect the hail resistance.

## 1. Introduction

**Figure 1:**
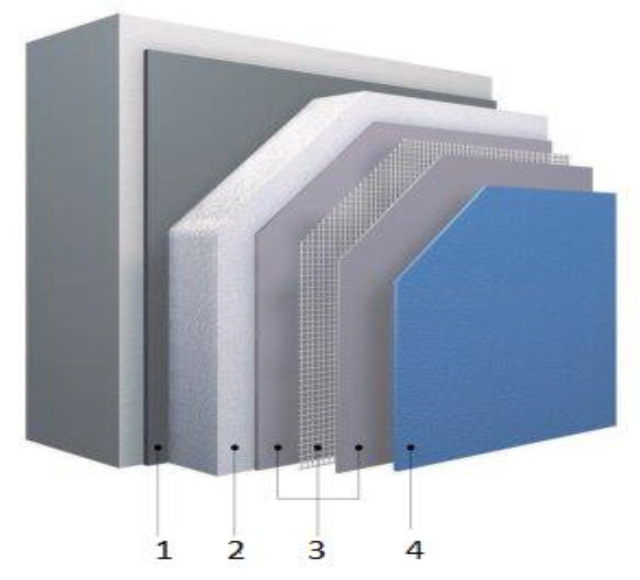
External thermal insulation composite systems (ETICS) components: 1—adhesive, 2—thermal insulation material (it can be fixed with mechanical connectors-pins), 3—reinforcement (mortar + glass fiber mesh), 4—finishing coat.

In recent years, damage caused by more frequent hail storm occurrences [1,2] are very important for durability of facade insulation systems. Hail induced depressions and cracks are not only an optical degradation, but also a functional damage of the protective render system [3,4,5,6]. Resulting water intake may particularly lead to biodeterioration, which can ruin the entire façade [7,8,9]. Currently, the durability of external thermal insulation composite systems (ETICS) and the moisture problem is receiving attention even in the Mediterranean area, which has a mild climate [10,11,12]. External thermal insulation composite system, commonly known as ETICS, is one of the most common solutions for building facades finishing (Figure 1).

The systems are applied to new and existing buildings and have been used worldwide since the end of 1950s [13,14,15]. They can be used on vertical walls made of masonry (bricks, blocks, stones, etc.) or concrete (cast on site or as prefabricated panels) and on horizontal or inclined surfaces that are not exposed to precipitation. ETICS are designed to give the wall to which they are applied to satisfactory thermal insulation. They should provide a minimal thermal resistance in excess of 1 m²K/W. The characteristics of the system and its components are described in the European Technical Approval Guidelines ETAG 004/2013 [16], while for the needs of domestic technical assessments, e.g., in Poland, they are included in WO-KOT/04/01 [17] and WO-KOT/04/02 [18]. The ETICS kit comprises prefabricated insulation product bonded onto the wall, or mechanically fixed using anchors, profiles, special pieces, etc., or a combination of adhesive and mechanical fixings. The insulation product is faced with a rendering consisting of one or more layers (site applied), one of which contains a reinforcement. The rendering is applied directly to the insulating panels, without any air gap or disconnecting layer. The ETICS may include special fittings (e.g., base profiles, corner profiles, etc.) to connect them to adjacent building structures (apertures, corners, parapets, etc.). The ETICS are non-load-bearing construction elements. They do not contribute directly to the stability of the wall on which they are installed. The ETICS can contribute to durability [19,20,21] by providing enhanced protection from the effects of weathering. An important issue related to the reference systems on building structures is their resistance to hail [7]. The research focused, among others, on the prediction of hail damage of buildings roofs and facades; therefore, research was conducted on hail characteristics (size, recurrence intervals, etc.) and hailstone impacts on building facades. The vertical and horizontal components of velocity of a hailstone were treated separately as well as measurements and predictions of the vertical velocity. Horizontal velocities of hailstones in gusty winds beneath thunderstorms were estimated [1]. Certain types of damage generated by the impact of a solid object were correlated with the amount of force 6 J that is localized around the area of contact between the 6 J impactor and the surface of the target [22,23]. Natural hail stones are described in the literature as spherical lumps with diameters of 0.5 to 10 cm (under extreme conditions even up to 18 cm) and weights of 0.1 to 500 g [24]. According to European Severe Weather Database, around 400 cases of large hail (hailstones having a minimum diameter of 2 cm) are reported in Poland annually [25]. Final velocities of hail stones before impact are in the range of 10 to 50 m/s leading to impact energies of 0.01 up to 100 J (in extreme events even up to 1000 J [24]). According to ETAG 004 [16], this feature is determined with a hard body impact method, e.g., a steel ball with a diameter of 50 mm and 500 g weight, and a ball with a diameter of 62.5 mm and one kilogram weight, whose impact energy amounts to three joules and ten joules, respectively. Another method is used in Switzerland and Austria to evaluate the characteristics, and involves hitting the ETICS surface with ice balls of various diameters at a 45° angle [8]. An attempt was made to compare the results of the tests obtained according to the two research methods. The analyses covered the impact dynamics, described by the impact energy, at a specific velocity and the depth and shape of the damage. It was shown that the 45° ice balls’ impact led to lower indentation depth and consequently to lower tensile strain and damage than 90° ice balls impact. However, surface parallel movement of the impactor was the cause of the formation of an elongated damage pattern in the 45° impacts [26].

The aims of this manuscript are twofold: first to present new, own method of assessment of the resistance of ETICS systems to hail; second: comparison of the results obtained according to this research method with the results obtained according to the methods described in ETAG 004. The basic premise of the presented new research methodology is evaluation of the thermal insulation system surface damage in the function of hit’ velocity with a polyamide ball with a standardized diameter and weight. It was assumed that the measure of resistance of the insulation system to impact is the ball speed at which no damage occurs. First test results according to the discussed method were already presented in scientific conferences [27]. In this manuscript there was made a comparison of hail resistance of various ETICS systems solutions depending on: first of all the type of adhesive used for reinforcement and additionally on the weight of the reinforcing mesh. The results of hail resistance obtained as a part of our own research method were compared with the values of hard body impact resistance obtained in tests performed according to methods written in ETAG 004.

## 2. Materials and Methods

### 2.1. Materials

The most common solution in Poland—expanded polystyrene (EPS) boards that comply with EN 13163 [28] with the perpendicular tensile strength of ≥ 100 kPa (EPS TR 100)—was selected for testing from among the thermal insulation materials. For the base coat reinforcement was selected a standard glass fiber mesh and for comparison armored glass fiber mesh. In the case of render, in research was used the most economical option, i.e., acrylic render.

So, the following materials were used in all tested systems:

- thermal insulation made of expanded polystyrene (EPS) with tensile strength perpendicular to the front planes ≥100 kPa, thickness of 50 mm,
- glass fiber mesh with tensile strength, after aging for 28 days in an alkaline environment (in accordance with ETAG 004/2013 [16] point 5.6.7.1.2), ≥20 N/mm, with the maximum reduction of this strength up to 50% in relation to the value before the action of aging factors; mass per unit area- 160 g/m^2^, mesh sizes: (3.5 × 3.5) mm
- acrylic rendering composed of sand, acrylic binder, silicone resin, mineral fillers, and additives; grain size: 1.5 mm, with bulk density 1.85 g/cm^3^.

The adhesive used for making the reinforcement, i.e., for immersing the mesh, was the variable component in the tested sets. For comparison, two types of adhesives were used in the tests, i.e.: cement–based dry mixture mixed with water and polymer dispersion. Used in research, three cement–based adhesives differed in bulk density, dry mixture consumption, and water amount. Polymer dispersion adhesive was treated comparatively as an alternative solution for cement-based adhesives. The sets composition was as follows:

- Set I: cement-based dry mixture mixed with water in the amount of 0.20 l/kg, density 1.40 g/cm^3^, ca. 98.9–99.9% ash content after roasting the mixture at temperature at 450 °C, dry mixture consumption of 3. 0 kg/m^2^, thickness (3.0–4.0) mm.
- Set II: polymer dispersion, mineral fillers, organic additives, density 1.70 g/cm^3^, dry matter content 70.2–85.8%, 69.2–76.4% ash content after roasting the mixture at temperature 450 °C and 64.5–71.3% after roasting the mixture at temperature 900 °C; consumption of 4.5 kg/m^2^, thickness (3.0–4.0) mm.
- Set III: cement-based dry mixture mixed with water in the amount of 0.30 l/kg, density 1.25 g/cm^3^, ca. 98.5–99.5% ash content after roasting the mixture at temperature at 450 °C, dry mixture consumption of 3.5 kg/m^2^, thickness (3.0–4.0) mm.
- Set IV: cement-based dry mixture mixed with water in the amount of 0.27 l/kg, density 1.40 g/cm^3^, 97.5–99.5% ash content after roasting the mixture at temperature at 450 °C; dry mixture consumption of ca. 4.0 kg/m^2^, thickness (3.0–4.0) mm.

For additional tests, made only for polymer dispersion adhesive, glass fiber mesh, called armored (for tests marked D), of mass per unit area of 330 g/m^2^ and mesh sizes: (6.6 × 8.9) mm were used. This mesh was also resistant to alkalis identified with the following parameters: breaking stress after ageing in an alkaline environment ≥20 N/mm and a decrease in the stress after ageing in comparison to the stress value of the material before the action of aging factors ≤50%.

For such additional tests the sets composition was as written below in Table 1.

### 2.2. Sample Preparation

The samples were made by hand in the form of mock-ups (0.5 × 1.0) m, from which, after conditioning, test samples with dimensions (200 × 200) mm or 200 mm diameter samples were cut out. Adhesive layer was applied with a notched trowel onto the thermal insulation layer, 3 mm thick in the case of using single mesh and 5 mm in the case of using double mesh, with stripes of the width of the reinforcing mesh. The glass mesh was immediately embedded in the adhesive. The surface of the adhesive layer was leveled with a smooth trowel. Finishing coat was applied after three days. Primer, which is part of the plastering system, was applied n the prepared surface and after another 24 h, the plaster. The samples were conditioned for 28 days in laboratory conditions, i.e., at a temperature (23 ± 2) °C and relative humidity 50 ± 5%.

### 2.3. Methods of Tests

The research methods used to assess hail resistance were divided into two groups. The first group is a new research method presented in this manuscript, the second uses the methodology adopted in ETAG 004. The purpose of the new research method was to create tools for more precise assessment of hail resistance than it is possible in accordance with the research methodology adopted in ETAG 004/2013 [16].

#### 2.3.1. Resistance to Hail

Resistance to hail performed according to the new research method [29], was conducted using the equipment described in PN-EN 13583:2012 [30]. The following research assumptions were made:

- the test samples represented the actual arrangement of the render coating,
- the test was carried out at (23 ± 2) °C, i.e., with no additional cooling of the sample surface,
- hail impact was simulated with a polyamide ball with a (40 ± 0.5) mm diameter and (38.5 ± 0.5) g weight, with a smooth surface, by hitting the sample surface on the plaster side at a 90° angle against the evaluated set plane, at a variable velocity. The speed causing damage to the sample was determined by the method of successive approximations, changing the speed of the ball every 1 m/s, starting from 5 m/s,
- the maximum velocity at which no damage/puncture of the render coating occurred was the test result, as the mean values obtained for 10 tested samples.

**Figure 2:**
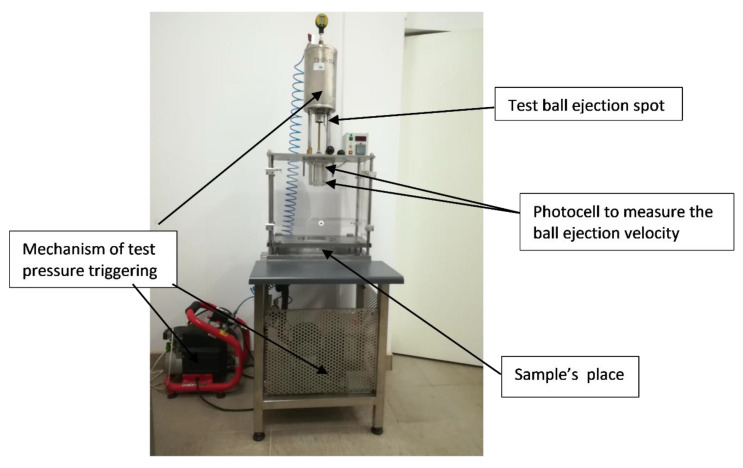
Equipment for hail resistance test.

The test equipment is shown in Figure 2.

**Figure 3:**
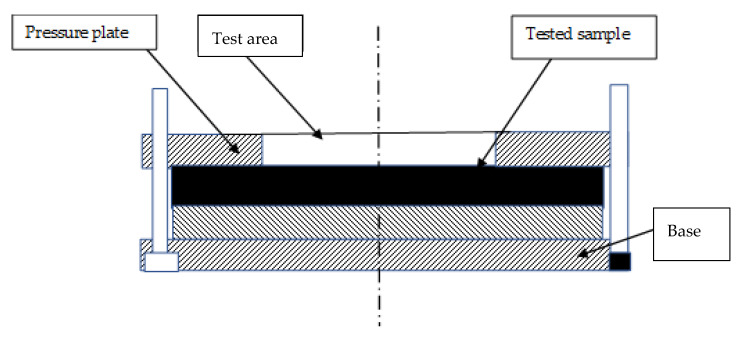
Test rig cross-section.

Samples of the thermal insulation sets (0.5 × 1.0) m were seasoned for 28 days in laboratory conditions (i.e., (23 ± 2) °C temperature and 50% ± 5% relative humidity). Then, 200 mm diameter samples were cut out from the main samples and after 24 h of seasoning in the abovementioned conditions they were placed on the equipment base with the plaster-coated face up and loaded with a pressure plate. The arrangement of the sample is shown in Figure 3.

The test started at a velocity at which puncture was expected (determined by the method of successive approximations as written above), and when the value was hard to determine, at the maximum achievable velocity of the equipment, i.e., 36 m/s. If no damage to the sample occurred at the velocity, the test was deemed as finished. If the sample broke, the test was repeated on other samples until the value that did not cause any damage was identified. The first visible plaster crack at the place where the test ball hits is considered as damage. The results of all tests were recorded, including the ball velocity and the kind of damage observed. Additionally, the velocity was converted to kinetic energy of the impact, which does not cause damage, according to the formula:

E_k_ = ½ m v^2^ (1)

where: m is polyamide ball weight, v is maximum ball velocity that does not puncture the sample.

#### 2.3.2. Resistance to Hard Body Impact

Hard body impact resistance was tested according to ETAG 004/2013 method [16], using samples of thermal insulation systems described in Section 2.1. The test was carried out after 7 days of exposure to water, followed by the samples conditioning in laboratory conditions, i.e., (23 ± 2) °C temperature and 50% ± 5% relative humidity. The test involved hitting the samples with steel balls weighing 0.5 kg and 1.0 kg with the impact energy of 3 J and 10 J, respectively. The result of the test was the specific value of the impact.

## 3. Results

This paragraph presents the results of the research, which was done in order to confirm the usefulness of the developed research method and answer the question of which of the components of ETICS has the greatest impact on resistance to hail impact, i.e., is it the type of adhesive used in the reinforcement or the numbers of meshes.

**Figure 4:**
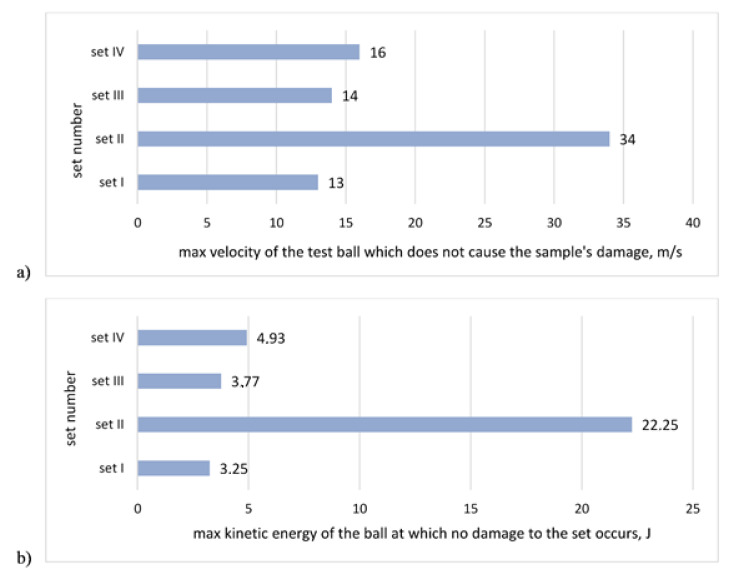
Results of hail resistance tests for sets with different adhesives and the same mesh: (**a**) in reference to the maximum velocity of the test ball, which does not cause the sample’s damage, (**b**) calculated as the maximum kinetic energy of the test ball, which does not cause the sample damage.

**Figure 5:**
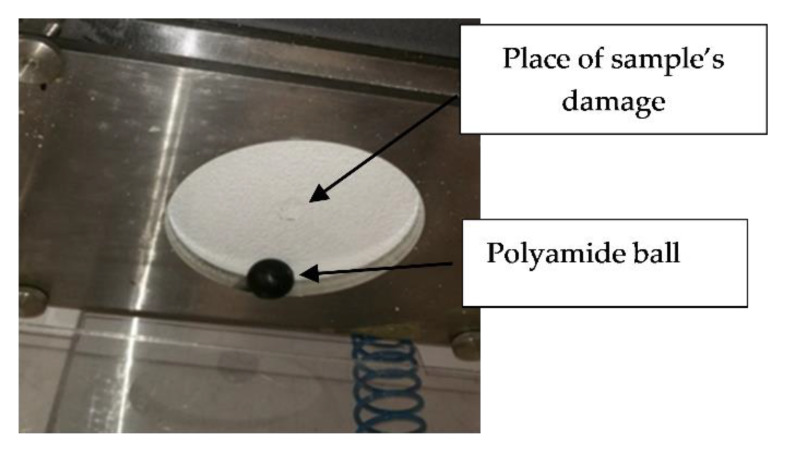
Sample after hail resistance test with a visible mechanical damage in the middle.

The test results of hail resistance tests in reference to the maximum impact velocity that does not cause the sample puncture for sets with the same single mesh are shown in Figure 4a, while Figure 4b shows the results calculated as per maximum kinetic energy of the ball. Figure 5 shows the sample after a test. In all examined cases, the damage was of a similar nature, i.e., the render crack was discovered at the boundary of the impact spot.

**Figure 6:**
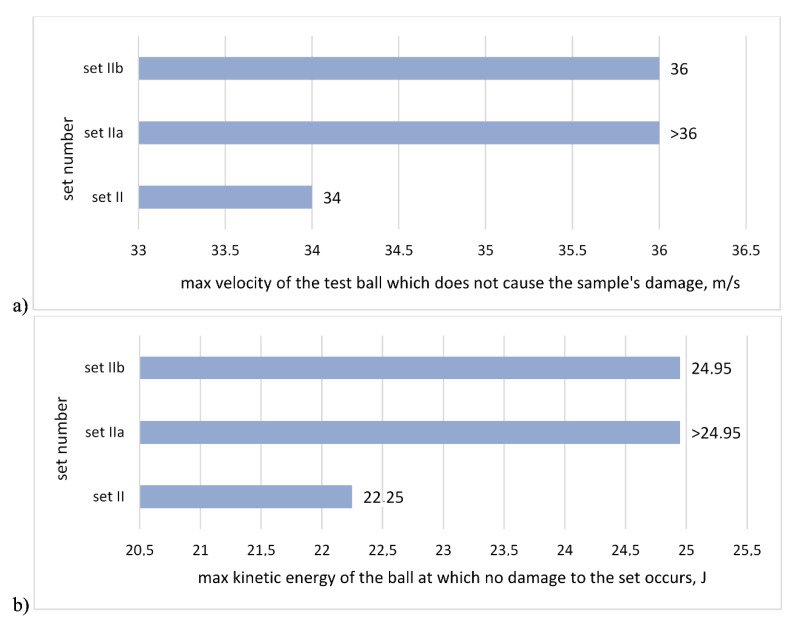
Results of hail stone puncture resistance tests for sets with different meshes and the same polymer dispersion adhesive: (**a**) in reference to the maximum velocity of the test ball which does not cause the sample’s damage, (**b**) calculated as the maximum kinetic energy of the test ball, which does not cause the sample’s damage.

The test results of hail puncture in reference to the maximum hit velocity, which does not cause the sample’s puncture for sets with combination of double meshes and the same polymer dispersion adhesive are shown in Figure 6a, while Figure 6b shows the results calculated as per maximum kinetic energy of the ball.

The test results of hard body impact resistance according to ETAG 004/2013 [16] are presented in Table 2.

## 4. Discussion

**Figure 7:**
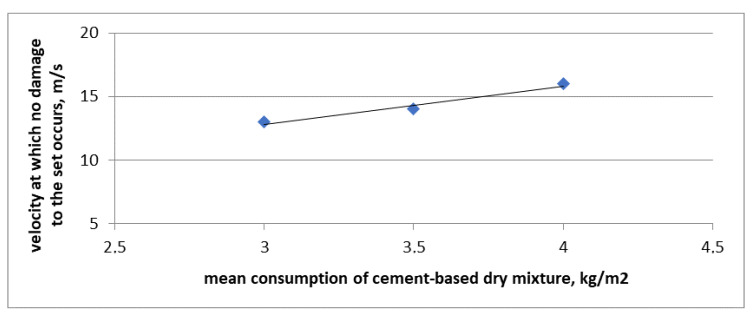
Comparative specifications of resistance to hail puncture and consumption of cement-based dry mixture used for making the reinforcement layer.

The obtained results of hail impact resistance are of a comparative nature because the puncture was simulated at a straight angle against the facade plane. In the building practice, the standard impact angle of hail stone is usually less than 90°. The actual angle of the hail stone hitting the facade depends on the building structure location, climate zone in which the building is situated, its height, wind force, impact direction, etc. Taking into account the complexity of the subject matter under natural conditions and consequently no repeatability or reproducibility of the test results suffering from the abovementioned influences, it was decided that the most favorable approach to the issue evaluation is to use the methods that enable comparing the obtained results. Hence a decision to use a straight angle in the hail impact simulation test. The obtained results suggest that the type of adhesive used for reinforcement, i.e., for gluing the mesh, greatly influences the resistance of the set to hail. Cement-based adhesives reveal resistance to puncture by the reference ball, at the maximum impact velocity from 13 m/s to 16 m/s, but no regularity of the obtained values was observed, depending on the mixing ratio of the powder ingredient with water. As the consumption of cement-based mixture goes up, the velocity required to damage the set also increases. In this case a nearly linear relationship can be observed. It is shown in Figure 7. The straight line in Figure 7 marks the linear trend relating to the obtained results.

The most favorable values for samples with cement-based adhesives were obtained for set IV, at the dry mixture consumption of 4.0 kg/m^2^, for which no damage was observed at the velocity of 16 m/s. The velocity corresponds to the kinetic energy of the ball of ca. 5 J. In the same material group, for the presented system, the most favorable results were also obtained in the hard body impact test. Two out of five tested samples were not damaged even when hit with 10 J energy.

Based on the comparison of the results it can be inferred that the cement-based dry mixture consumption over 4.0 kg/m^3^ can contribute to the change in the thermal insulation system puncture resistance class.

**Figure 8:**
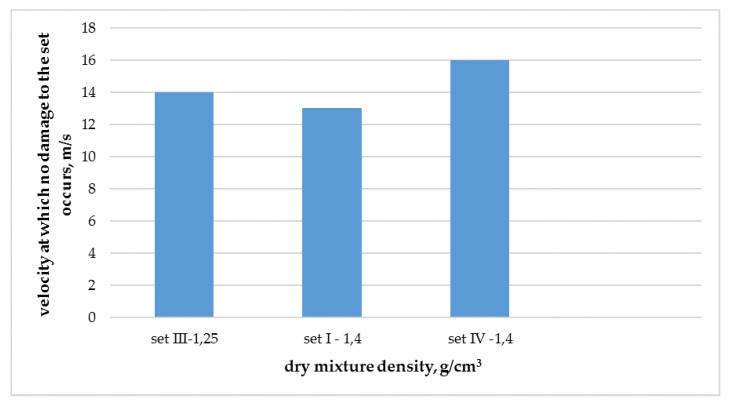
Comparative specifications of resistance to hail puncture and density of cement-based dry mixture used for making the reinforcement layer.

It is known in the literature that mechanical properties increase with increase of density. Unfortunately, with regard to hail resistance of thermal insulation composite systems with rendering, such a regularity is not fully confirmed, as shown in Figure 8. A clear deviation from this regularity is visible in the case of set I. It can be presumed that the lack of this correlation may be due to too small population of analyzed results.

**Figure 9:**
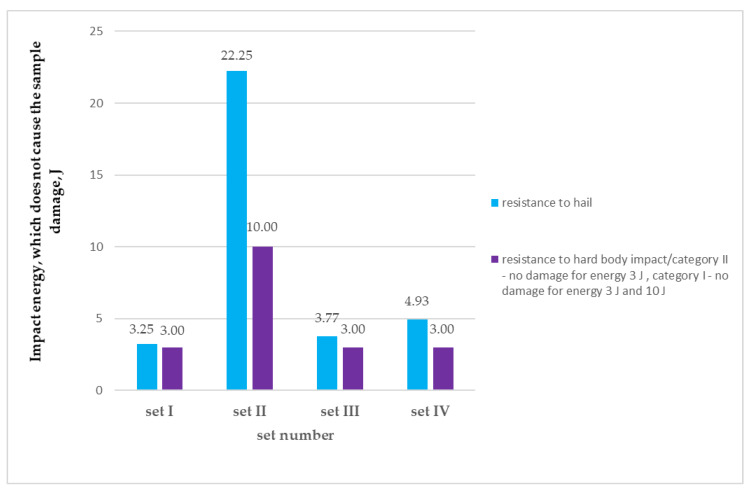
Comparison between results of hail resistance test and resistance to hard body impact for ETICS with different adhesives.

Although the method of hard body puncture testing is meant to simulate the hit on the facade of hard bodies with a fixed (irreversible) shape or with sharp edges which can incidentally impact the thermal insulation system, hail stones can be considered as items compliant with the abovementioned assumption in exceptional cases. According to ETAG 004 [16] the hit energy of ca. 10 J is the limit value for classifying a thermal insulation system into the highest puncture resistance category, i.e., class I. The class is achieved when no damage occurs as a result of hit with 10 J and 3 J energy. If the evaluated set meets only one of the abovementioned requirements, i.e., no cracks occur as a result of hit with 3 J energy, it can be identified as class II. The assumed evaluation categories in hard body puncture method do not include possibilities of intermediate classification, i.e., hit energy between 3 J and 10 J, which is possible according to the new method of testing resistance to hail. The evaluation system assumed in ETAG 004 [16] understates the classification of many products and assigns class II to them, whereas they often transfer loads much higher than 3 J. It is true for set IV for which the achieved values of resistance to hail puncture amounted to 4.93 J. A significant increase in the puncture resistance as a result of simulated hail influence and hard body impact was observed for the set with dispersion adhesive (set II), which was confirmed by the previous test results for this group of products [8]. The velocity of the ball that causes damage to the set is four times higher than for cement-based adhesives. The maximum kinetic energy of the ball which does not cause damage to the set, in the hail resistance test, amounted to ca. 22.25 J, which twice exceeds the values which can be evaluated according to the hard body impact test method based on ETAG 004 [16], i.e., 10 J. Despite the fact that the system with dispersion adhesive was included in the highest puncture resistance category in the hard body impact test, the test result does not reflect the actual and very high resistance to such a hit. In addition, results obtained for both research methods are graphically compared in Figure 9.

Mass per unit area of the reinforcing mesh used in the system is not as significant, as expected, to affect to hail resistance of the system (Figure 6). Surprisingly, the result of hail resistance for ETICS systems with two layers of 160 g/m^2^ mesh was higher than for system with armor mesh and 160 g/m^2^ mesh. Better values were obtained using a double mesh system, each with a weight of 160 g/m^2^, than in the case of a combination of armor and 160 g/m^2^ mash. In the case of combination of armor and traditional mesh, no puncture was obtained only for three out of ten tested samples, at 36 m/s hit velocity, while in the case of a double mesh with a weight of 160 g/m^2^, none of the ten tested samples were broken at a ball velocity of 36 m/s. It has not been determined yet what ball velocity causes damage to this test set, because the velocity of 36 m/s is the maximum value that can be obtained in the device. It is only possible to say that the application of the additional layer of the same mesh caused an increase in hail resistance to a value exceeding 25 J. The use of the armored glass fiber mesh, together with the standard mesh, allowed to increase the resistance of the ETICS to the same value, i.e., 25 J.

The performed tests did not help to determine direct correlation of the results obtained with the two presented test methods, i.e., hail impact resistance and hard body impact resistance, but significant similarities were discovered. The results obtained with both methods are highly similar, after taking the measurement differences into account, i.e., using a ball made of material with different hardness and rigidity, different ways of applying the force, etc. Estimation of the measurement uncertainty value will be possible after completing a higher number of comparative studies.

## 5. Conclusions

The paper presents test results of four random ETICS sets. With regard to the small population of the test objects, the results cannot be generalized but they should be treated as significant symptoms which require further studies. Taking the above into account, the following conclusions can be drawn:

➢ There is a relationship between the results of puncture resistance tests obtained according to the hail puncture resistance test method presented in this manuscript and hard body impact test according to the method described in ETAG 004 [16].
➢ Hail puncture resistance tests help to evaluate precisely the resistance of thermal insulation sets for damage as a result of a hit of heavy objects of permanent (non-deformable) shape, or sharp edges, with greater accuracy than the hard body impact test.
➢ Thermal insulation system sets with dispersion adhesive in the reinforcement demonstrate greater resistance to damage as a result of hail hit than the sets with cement-based adhesives.
➢ Mass per unit area of the reinforcing mesh used in the system is not significant to affect to hail resistance of the system.

## Figures and Tables

**Table 1:** Configuration of thermal insulation systems for additional sets.

Set no	Insulation Product	Base Coat	Glass Fiber Mesh		Mesh Sizes, mm	Finishing Coat
Kind of Mesh	Total Mass per Unit Area, g/m^2^
Set IIa	characterized above as A	polymer dispersion characterized above in set II	With double mash characterized above as B, i.e.,: 2 × B	320	First and second layer: 3.5 × 3.5	characterized above as C
Set IIb			with combination of mashes, i.e.,: characterized above as B + D	490	First layer: 3.5 × 3.5; Second layer: 6.6 × 8.9	

**Table 2:** Results of hard body impact resistance test, according to ETAG 004/2013 [16].

Set Number	Tests Results after	
	Impact 3 Joule	Impact 10 Joule
Set I	no deterioration of 5 tested samples	➢ Cavity without deterioration of 1 tested sample; ➢ circular deterioration of 3 tested samples, diameters: 3.6 mm, 4.0 mm, 3.1 mm, ➢ one crack length of 1 cm
Set II	no deterioration of 5 tested samples	no deterioration of 5 tested samples
Set III	no deterioration of 5 tested samples	➢ Cavity without deterioration of 2 tested samples; ➢ circular deterioration of 3 tested samples about diameters: 3.1 mm, 3.2 mm and 3.5 mm
Set IV	no deterioration of 5 tested samples	➢ Cavity without deterioration of 3 tested samples; ➢ circular deterioration of 2 tested samples about diameters: 3.1 mm, and 3.6 mm
